# GPER agonist G-1 decreases adrenocortical carcinoma (ACC) cell growth *in vitro* and *in vivo*

**DOI:** 10.18632/oncotarget.4241

**Published:** 2015-06-05

**Authors:** Adele Chimento, Rosa Sirianni, Ivan Casaburi, Fabiana Zolea, Pietro Rizza, Paola Avena, Rocco Malivindi, Arianna De Luca, Carmela Campana, Emilia Martire, Francesco Domanico, Francesco Fallo, Giulia Carpinelli, Lidia Cerquetti, Donatella Amendola, Antonio Stigliano, Vincenzo Pezzi

**Affiliations:** ^1^ Department of Pharmacy, Health and Nutritional Sciences, University of Calabria, Arcavacata di Rende, Cosenza, Italy; ^2^ Department of Medicine-DIMED, University of Padova, Padova, Italy; ^3^ Department of Cell Biology and Neurosciences, National Institute of Health, Rome, Italy; ^4^ Department of Clinical and Molecular Medicine, Sant'Andrea Hospital, Faculty of Medicine and Psychology, Rome, Italy; ^5^ Research Center, San Pietro Hospital-Fatebenefratelli, Rome, Italy

**Keywords:** GPER, G-1, adrenocortical cancer, apoptosis

## Abstract

We have previously demonstrated that estrogen receptor (ER) alpha (ESR1) increases proliferation of adrenocortical carcinoma (ACC) through both an estrogen-dependent and -independent (induced by IGF-II/IGF1R pathways) manner. Then, the use of tamoxifen, a selective estrogen receptor modulator (SERM), appears effective in reducing ACC growth *in vitro* and *in vivo*. However, tamoxifen not only exerts antiestrogenic activity, but also acts as full agonist on the G protein-coupled estrogen receptor (GPER). Aim of this study was to investigate the effect of a non-steroidal GPER agonist G-1 in modulating ACC cell growth. We found that G-1 is able to exert a growth inhibitory effect on H295R cells both *in vitro* and, as xenograft model, *in vivo*. Treatment of H295R cells with G-1 induced cell cycle arrest, DNA damage and cell death by the activation of the intrinsic apoptotic mechanism. These events required sustained extracellular regulated kinase (ERK) 1/2 activation. Silencing of GPER by a specific shRNA partially reversed G-1-mediated cell growth inhibition without affecting ERK activation. These data suggest the existence of G-1 activated but GPER-independent effects that remain to be clarified. In conclusion, this study provides a rational to further study G-1 mechanism of action in order to include this drug as a treatment option to the limited therapy of ACC.

## INTRODUCTION

Adrenocortical carcinoma (ACC) represents a rare malignancy with a very poor prognosis. Resectability is the prime determinant of prognosis. For patients with disseminated disease, chemotherapy options are few and lack sufficient efficacy. Mitotane, a cytotoxic drug with a not well documented mechanism of action [1], is the conventional therapy. The toxicity of mitotane has been a major limit to its suitability in the treatment of ACC patients. Severe side-effects, of either the gastrointestinal or the nervous system, have been frequently reported, and many patients are not able to take the drug regularly [2, 3]. Recently, monoclonal antibodies targeting insulin-like growth factor (IGF) receptor (IGF1R) have been tested in clinical trials, however, they provided a limited effectiveness in refractory patients [4]. Rationale for targeting IGF1R comes from the observation that IGFII [5] is overexpressed in ACC. IGFII effects are mediated through its receptor IGF1R resulting in activation of the PI3K/AKT/mTOR cascade, the RAS/MAPK and the PLC/PKC pathways [6]. We have recently demonstrated that activation of these pathways can be triggered by the estrogen receptor alpha (ESR1) [7], a gene overexpressed in ACC that mediates estrogen-dependent proliferative effects [7, 8]. Our *in vitro* experiments demonstrated that ESR1 knock down was more effective than an IGF1R antibody in controlling H295R cell proliferation [7]. Targeting ESR1 *in vivo* using tamoxifen, a selective estrogen receptor modulator (SERM), was effective in reducing H295R xenografts growth [7].

It is well known that tamoxifen and its active metabolite 4-hydroxytamoxifen (OHT), not only exert antiestrogenic activity [9], but also act as full agonist on the G protein-coupled estrogen receptor GPR30 (from the GPER gene) [10–14]. Then, can Tamoxifen effects depend on GPER activation? GPER can mediate rapid E2-induced non-genomic signaling events, including stimulation of adenylyl cyclase, mobilization of intracellular calcium (Ca^2+^) stores and activation of mitogen-activated protein kinase (MAPK) and phosphoinositide 3-kinase (PI3K) signaling pathways [15–17]. GPER exhibits prognostic utility in endometrial [18], ovarian [19], and breast cancer [20] and can modulate growth of hormonally responsive cancer cells [10, 11, 21, 22]. Expression of GPER has been characterized in the outer zona glomerulosa (ZG) and in the medulla of the human adrenal [23], however its expression status in ACC is not known.

A non-steroidal, high-affinity GPER agonist G-1 (1-[4-(6-bromobenzo [1, 3]dioxol-5yl)-3a, 4, 5, 9b-tetrahydro-3H-cyclopenta-[c]quinolin-8-yl]-ethanone) has been developed to dissect GPER-mediated estrogen responses from those mediated by classic estrogen receptors [24]. The biological effects triggered by G-1 appear cell type specific and dependent on the ERs expression pattern [25–29]. By using G-1, in this study we wanted to investigate the effects of GPER activation on ACC growth.

## RESULTS

### G-1 treatment decreases H295R cell growth *in vitro* and *in vivo*

**Figure 1:**
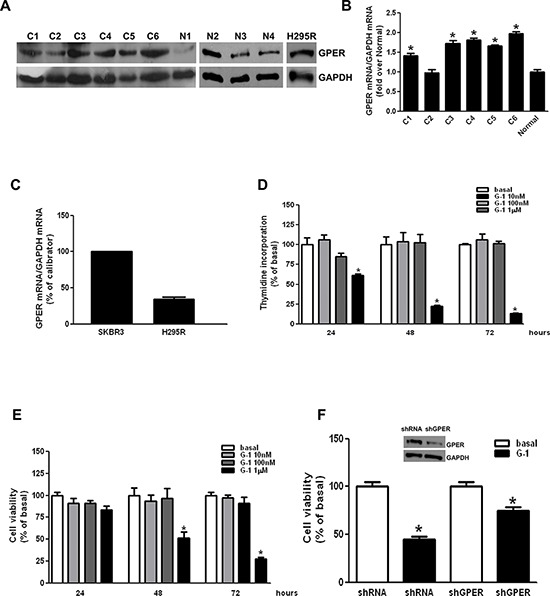
G-1 treatment decreases H295R cell growth *in vitro* **A.** Western blot analysis of GPER was performed on 50 μg of total proteins extracted from normal adrenal, ACCs and H295R cells. GAPDH was used as a loading control. **B-C.** GPER mRNA expression in normal adrenal and ACCs (B), H295R and SKBR3 (positive control) cells (C) was analyzed by real time RT-PCR. Each sample was normalized to its GAPDH RNA content. Final results are expressed as n-fold differences of gene expression relative to calibrator. Data represent the mean + SE of values from at least three separate RNA samples; *P < 0.05, versus calibrator). **D-E.** H295R cells were treated with G-1 (0.01–1 μM) for different times (24, 48 and 72 h). Cell proliferation was evaluated by [^3^H]Thymidine incorporation (D) and MTT (E) assays. Results were expressed as mean + SE of three independent experiments each performed in triplicate. Statistically significant differences are indicated (*P < 0.05 versus basal). **F.** MTT assay was performed on H295R cells, which were previously transfected for 72 h in the presence of control vector (shRNA) or shGPER. Twenty-four hours after transfection cells were treated in 2.5% DCC-FBS medium for 48 h with G-1 (1 μM). Results were expressed as mean + SE of three independent experiments each performed in triplicate. (*p < 0.05 versus basal). The insert shows a Western blotting assay on H295R protein extracts evaluating the expression of GPER receptor in the presence of shRNA or of shGPER. GAPDH was used as a loading control.

We first examined GPER expression in human ACCs and in H295R cells. By western blot analysis (Fig. 1A) and real time RT-PCR (Fig. 1B–1C) we demonstrated that GPER is expressed in normal adrenal, in human ACCs and in H295R cells at variable levels. Effects of G-1 on cell viability and proliferation were tested using increasing concentrations (0.01-0.1-1 μM) for different times (24-48-72 h) (Fig. 1D–1E). Of the different doses tested only 1 μM caused a time-dependent reduction in H295R cell growth. Doses higher than 1uM did not show any more pronounced effect (data not shown). Knocking down of GPER gene expression, using a specific shRNA, (shGPER) was assessed by western blot analysis and revealed a substantial decrease in protein content compared to the control shRNA (insert, Fig. 1F). However, GPER silencing was able to only partially abrogate the inhibitory effects exerted by G-1 on H295R cell proliferation (Fig. 1F)

**Figure 2:**
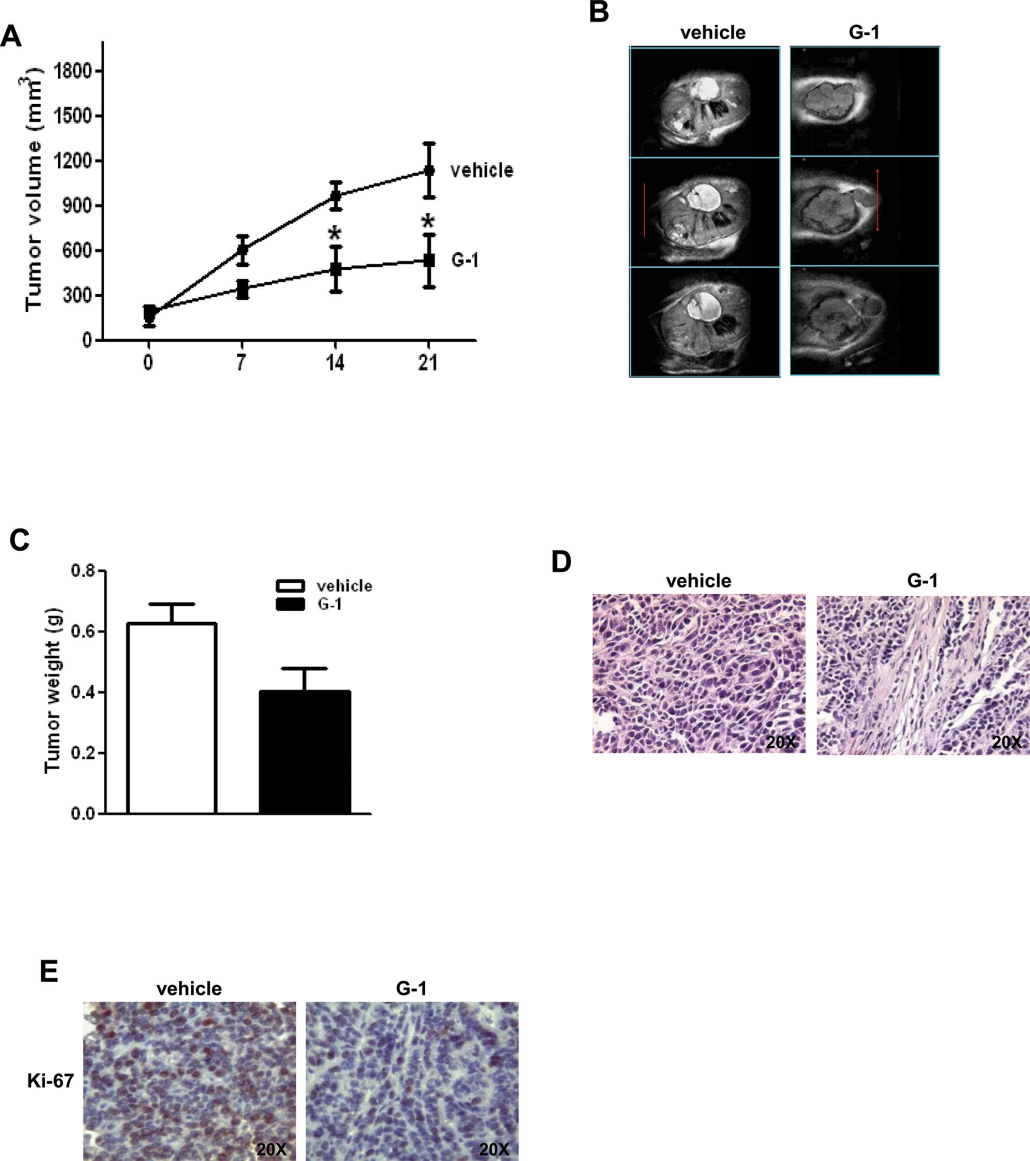
G-1 treatment decreases H295R cell growth *in vivo* **A.** 6 × 10^6^ H295R cells were injected subcutaneously in the flank region of immunocompromized mice and the resulting tumors were grown to an average of 200 mm^3^ twenty one days after inoculation. Tumor volumes were calculated, as indicated in Materials and Methods. Values represent the mean + SE of measured tumor volume over time in the control group (filled circles, *n* = 10) and in the G-1-treated group (filled triangles, *n* = 10). Data represent pooled values from two independent experiments. (*P < 0.05 versus control at the same day of treatment). **B.** *In vivo* coronal T2-weighted spin-echo MR image of primary ACCs. Examples of multi-slices T2-W MRI (section thickness of 1 mm) tumors from vehicle treated mice (control tumors) show a larger volume compared to tumors from G-1 treated mice. Hyperintense large cystic area and haemorrhagic regions that appear as dark areas in the tumor sections, are present in the control tumors. **C.** After 3-week treatment tumors were harvested and weighed. Values represent the mean + SE of measured tumor weight (*n* = 10) (* *P* < 0.05 versus vehicle). **D.** Hematoxylin and eosin stained histologic images of H295R xenograft tumors. **E.** Representative pictures of Ki-67 immunohistochemical staining of H295R xenograft tumors.

H295R cells were used to generate xenograft tumors in athymic nude mice. Twenty one days after tumor grafting all mice developed a detectable tumor and were randomized to be treated with either vehicle or G-1. G-1 administration produced a statistically significant decrease in tumor volume from day 14 post treatment (Fig. 2A). A trend of growth inhibition was observed thereafter. The drug was well tolerated without lethal toxicity or body weight loss during treatment (data not shown). Multi-slices T2-W MRI indicated larger tumor volume in vehicle treated animals compared to tumors from G-1 treated mice. Hyperintense large cystic area and haemorrahagic regions, that appear as dark areas in the tumor sections, were present in vehicle treated animals (Fig. 2B). Grafted tumors harvested after three-week treatment with G-1 showed a significant decrease in tumor weight compared to vehicle treated animals (Fig. 2C). Hematoxylin and eosin staining of xenograft tumors revealed some picnotic nuclei only in G-1 treated tumors (Fig. 2D). Ki-67 immunostaimning was significantly lower in G-1-treated tumors compared to control mice (value score control: 6, 6 ± 0, 89 (SD); value score G-1 treated cells: 3, 1 ± 0.55 * (SD) (*p < 0.05) (Fig. 2E).

### G-1 induces H295R cell cycle arrest and cell death

**Figure 3:**
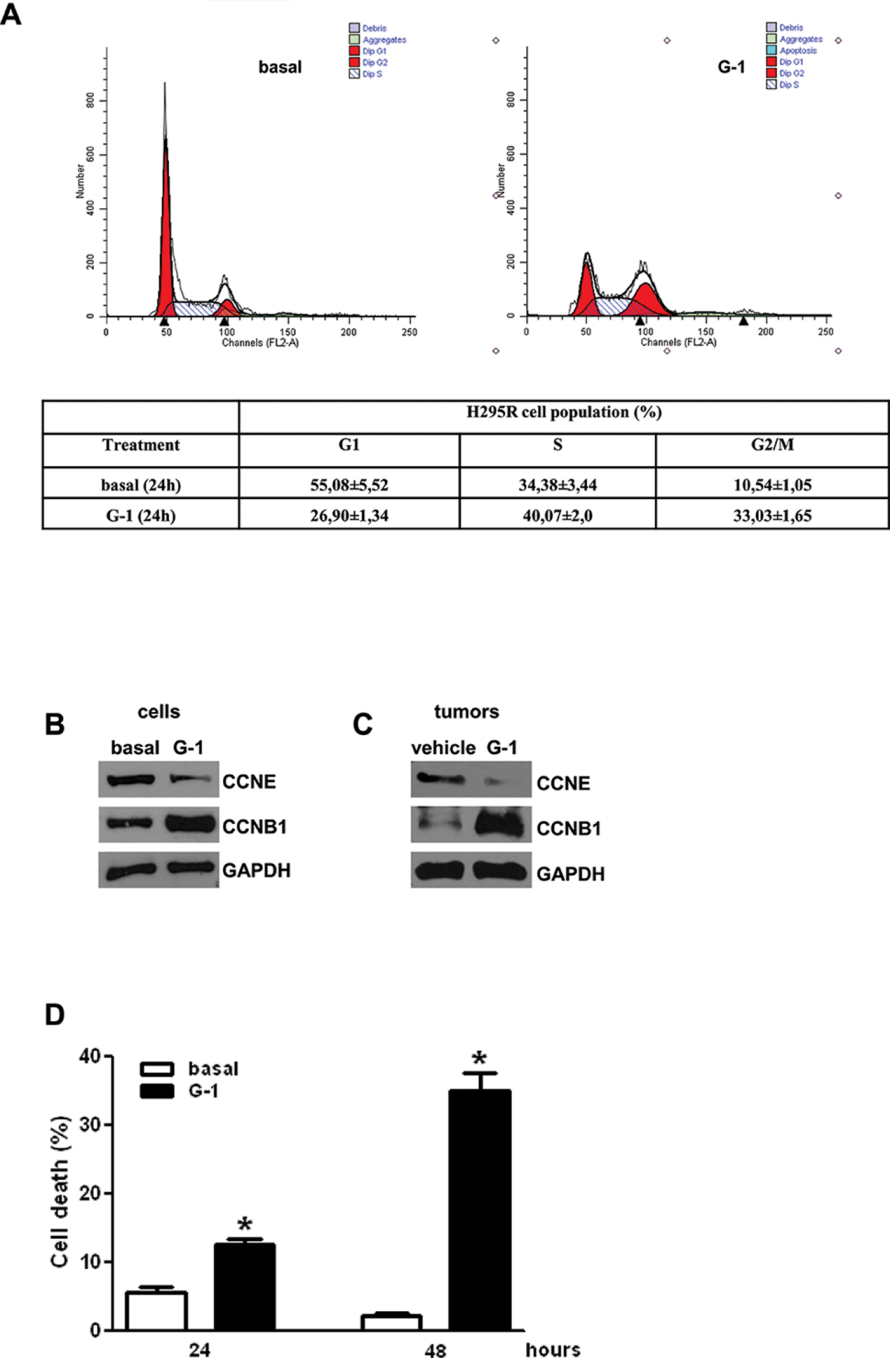
Effects of G-1 treatment on cell cycle distribution and on cell death **A.** H295R cells were synchronized in serum-free media for 24 h and then exposed to vehicle (basal) or G-1 (1 μM) for the indicated times. The distribution of H295R cells in the cycle was determined by Flow Cytometry using Propidium Iodide stained nuclei. Table shows the distribution of H295R cell population (%) in the various phases of cell cycle. **B-C.** Western blot analyses of Cyclin E (CCNE) and Cyclin B1 (CCNB1) were performed equal amounts of total proteins extracted from H295R cells treated with G-1 (1 μM) for 24 h (B) and xenografts tumors (C) Blots are representative of three independent experiments with similar results. GAPDH was used as a loading control. **D.** Subconfluent H295R monolayers starved for 24 h were treated for the indicated times with G-1 (1 μM). Then cells were stained with Annexin V/ FITC plus PI and examined by flow cytometer. Graph represents the percentage of cell death at the different times of treatment. (* *P* < 0.05 versus basal).

Cell cycle analysis of H295R cells after 24 h of G-1 treatment demonstrated a cell cycle arrest in the G_2_ phase (Fig 3A). This effect was further confirmed by a change in the expression of cyclins, after G-1 treatment (Fig. 3B). Specifically, by western analysis we observed that G-1 treatment caused a decrease in Cyclin E (CCNE), while Cyclin B1 (CCNB1), involved in the regulation of G_2_ phase, was increased. CCNE and CCNB1 had similar expression pattern in protein samples extracted from xenografts tumors (Fig. 3C). Collectively these events support the idea of cells exiting G_1_ but remaining stuck in G_2_ phase. In agreement with the observation that inappropriate accumulation of B type cyclins is associated with the initiation of apoptotic pathways [30], we found that G-1 caused cell death by apoptosis. Cells were treated for 24 or 48 h with vehicle or G-1, incubated with an Annexin-V specific antibody and sorted by flow cytometry. As shown in Figure 3D the number of dead cells increased in a time dependent manner reaching about 40% of apoptotic cells 48 h after G-1 treatment (Fig. 3D).

### G-1 causes cell nuclei morphological changes, DNA damage and apoptosis

**Figure 4:**
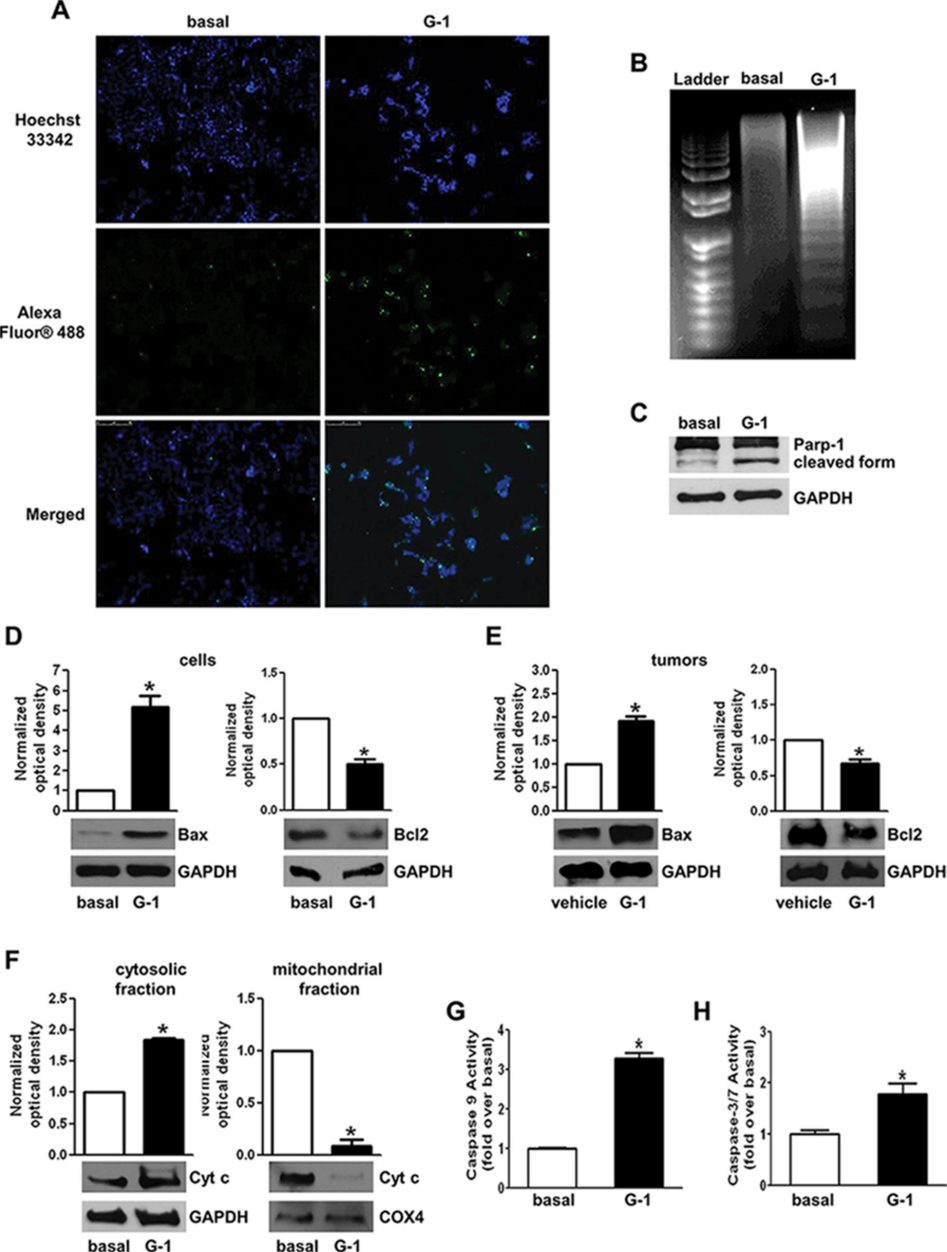
G-1 treatment induces apoptosis in H295R cells **A.** Cells were left untreated (basal) or treated with G-1 (1 μM) for 24 h; after treatment cells were fixed with paraformaldehyde and processed for TUNEL staining. Nuclei counterstaining was performed using Hoechst 33342. Fluorescent signal was observed under a fluorescent microscope (magnification 200X). Images are from a representative experiment. **B.** After 48 h treatment DNA was extracted from cells and analyzed on a 1.5% agarose gel. Images are from a representative experiment. **C–F.** H295R cells were treated with G-1 (1 μM) for 24 h. Western blot analyses of Parp-1 (C), Bax and Bcl-2 (D). Cytochrome c (F) were performed on equal amounts of total proteins. Blots are representative of three independent experiments with similar results. Bax and Bcl-2 were analyzed on total proteins extracted from xenograft tumors (E). GAPDH was used as a loading. **G-H.** H295R cells were treated with G-1 (1 μM) for 24 h. Caspase 9 (G) and caspase 3/7 (H) activity was determined by a luminescent assay. Results were expressed as percentage of enzyme activity. Graphs represent mean + SE of three independent experiments each performed in triplicate. Statistically significant differences are indicated (*P < 0.05 versus basal).

G-1 ability to trigger apoptosis in H295R cells was further confirmed by evaluation of DNA fragmentation. TUNEL staining demonstrated the presence of increased positive cells in cells treated with G-1 (Fig. 4A). In addition, Hoechst staining evidenced that untreated H295R cells had round nuclei with regular contours; while nuclei from cells treated with G-1 appeared shrunken and irregularly shaped or degraded with condensed DNA. DNA gel electrophoresis extracted from G-1 treated H295R cells revealed a classic laddering pattern of inter-nucleosomal DNA fragmentation that was absent in control cells (Fig. 4B). This event was associated with an increase in Parp-1 cleavage (Fig. 4C). The presence of G-1 increased Bax expression while decreased Bcl-2 (Fig. 4D). Similarly, data obtained from western blot analysis of tumors samples overlap with those obtained in H295R cells (Fig. 4E). When the intrinsic apoptotic mechanism is triggered, Cytochrome c (Cyt c) is released from the mitochondria into the cytosol [31]. Therefore we fractionated G-1 treated H295R cell lysates into cytosolic and mitochondrial fractions and evaluated Cytochrome c release by western blot analysis (Fig. 4F). Cytochrome c levels increased in the cytosolic fraction of treated samples while decreased in the mitochondrial compartment. Cytochrome c release from mitochondria into the cytosol triggers caspase activation. After G-1 treatment we detected active Caspase 9 (Fig. 4G) as well as the executioner Caspase 3/7 (Fig. 4H).

### G1 treatment causes sustained ERK1/2 phosphorylation

**Figure 5:**
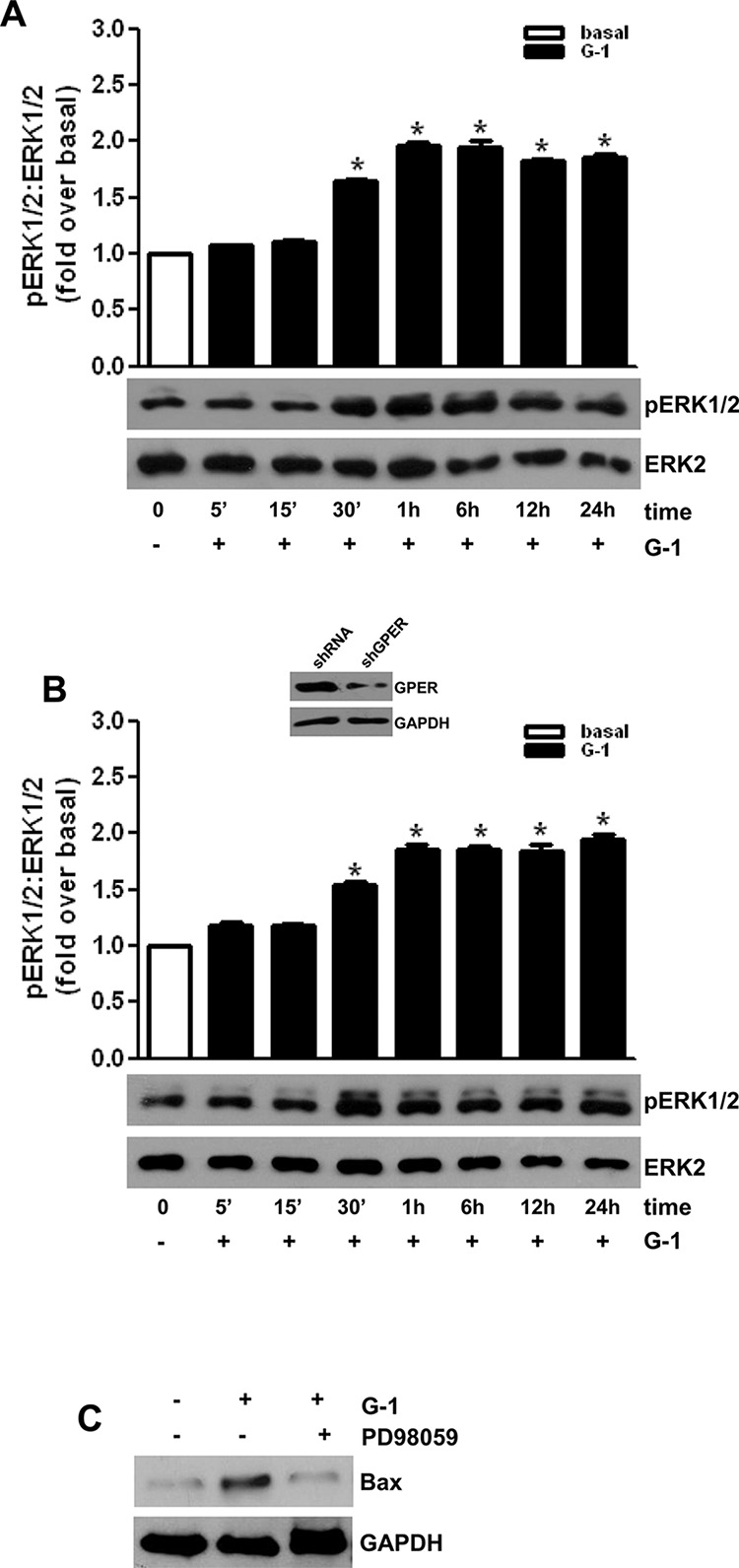
G-1-induced MAPK activation correlates with an increased protein expression of proapoptotic Bax H295R cells were transfected with shRNA **A.** or shGPER **B.** for 72 h. Forty-eight hours after transfection cells were untreated (0) or treated for at the indicated time with G-1 (1 μM). Western blot analyses of pERK1/2 were performed on 10 μg of total proteins. ERK1/2 was used as a loading control. Blots are representative of three independent experiments with similar results. The insert in (B) shows a Western blot on H295R protein extracts evaluating the expression of GPER receptor in the presence of shcontrol or of shGPER. GAPDH was used as a loading control. (A-B up panels) Graphs represent means of normalized optical densities from three experiments, bars represent SE. *p < 0.05 versus basal. **C.**, H295R cells were treated for 24 h with vehicle (−) or G-1 (1 μM) alone or combined with PD98059 (10 μM). Western blot analysis of Bax was performed on equal amounts of total proteins. GAPDH was used as a loading control. Blots are representative of three independent experiments with similar results.

In order to define the molecular mechanism associated with G-1-induced apoptosis, we investigated the activation of MAPK family members extracellular signal-regulated kinase 1/2 (ERK1/2), which have been demonstrated to be involved in apoptosis if activated for a prolonged time [32]. As shown in Figure 5A, G-1 treatment activated ERK1/2 in a time-dependent manner as seen by the increased levels of their phosphorylation status. Activation started after 30-min of G-1 treatment and persisted for up to 24 h (Fig. 5A). ShGPER, that partially reversed G-1 effects on cell proliferation (Fig. 1E) did not affect ERK1/2 activation (Fig. 5B). Involvement of ERK1/2 in G-1-induced apoptosis of adrenocortical cancer cells was confirmed by the observation that MEK1 inhibitor, PD98059, prevented the up-regulatory effect exerted by G-1 on Bax expression (Fig. 5C).

## DISCUSSION

Here, we demonstrated for the first time that a selective non estrogenic ligand of GPER named G-1 is able to inhibit H295R cell growth both *in vitro* and *in vivo* in a xenograft model. Starting from these results we investigated the potential role of GPER in this event.

First, we showed GPER expression both at transcriptional and post-transcriptional level in our ACC cell model represented by H295R cells as well as in normal adrenal and ACC samples. These first analyses aimed to assess only if GPER was expressed in normal and tumor adrenal and not to indicate any difference in expression levels, since overexpression of GPCR is not a common event in human diseases [20].

Recent studies have shown that activation of GPER initiates signaling cascades that, depending on the cell type, are associated with both proliferation [11, 33] and apoptosis [29, 32]. Ariazi et al. have highlighted the opposite effects played by GPER activation on cell proliferation of ERs negative and ERs positive breast cancer cells [17]. Specifically, when ERs are expressed, activation of GPER leads to inhibition of cell proliferation. On the contrary, when cells are ERs negative activation of GPER leads to an increase in cell proliferation [17]. Our work, demonstrated that micromolar concentrations of G-1 decrease H295R cell proliferation *in vitro*, significantly reduce ACC tumor volume *in vivo* and cause a marked decrease in the expression of the nuclear proliferation antigen Ki-67. Accordingly, flow cytometry analysis revealed that G-1 treatment causes changes in cellular distribution within the different phases of cell cycle. It is well established that cell cycle progression is dynamically and strictly regulated by complexes containing cyclins and cyclin dependent kinases (CDKs) [34]. Here, we found that after G-1 treatment expression of G_1_ phase cyclin CCNE was reduced, while G_2_ phase cyclin CCNB1 was increased. This observation indicates that H295R cells do not bypass G_2_ checkpoint. Similar data were reported for prostate cancer cells, where GPER activation by 1 μM G-1 caused cell cycle arrest at the G_2_ phase [35]. G_2_ phase arrest was followed by apoptotic cell death as indicated by positive staining for Annexin-V, nuclei morphological changes and appearance of DNA ladder pattern.

Apoptosis can be induced by extrinsic [36] and intrinsic [37] mechanisms; the latter is strictly controlled by bcl-2 family of proteins [38] that consists of both pro-(Bax, Bad, Bak, Bid) and anti-apoptotic (Bcl-2, Bcl-xl) proteins able to modulate the execution phase of the cell death pathway. Bax exerts pro-apoptotic activity by allowing Cytochrome c translocation from the mitochondria to the cytosol [39]. Cytochrome c then binds to apoptotic protease-activating factor-1 (Apaf-1) [40], which in turn associates with Procaspase 9 resulting in the activation of its enzymatic activity [41], responsible for the proteolytic activation of executioner Caspase 3 [42]. The active Caspase 3 is then involved in the cleavage of a set of proteins including Poly-(ADP) ribose polymerase-1 (Parp-1) [43]. Bcl-2, instead, exerts its anti-apoptotic activity, at least in part, by inhibiting the translocation of Bax to the mitochondria [40]. Changes in expression and/or activation of all the above mentioned biochemical markers of mitochondrial apoptotic pathway were observed in H295R cells in response to G-1 treatment.

MAPK family members ERK1/2 are part of GPER signaling [14]. Despite the well-defined role of ERK1/2 activation in proliferative pathways [44], sustained ERK1/2 phosphorylation is involved in apoptotic events [29, 32, 45]. Cagnol and Chambard have summarized more than 50 publications showing a link between prolonged ERK activation and apoptosis [46]. Specifically it can be appreciated that duration of ERK activation in promoting cell death can be different depending on cell type and stimuli. G-1 caused sustained ERK1/2 activation in H295R, this event was clearly involved in the induction of apoptosis, since chemical inhibition of MEK1/2 using PD98059 abrogated G-1 ability to induce the expression of proapoptotic factor Bax. Several reports pointed out that ERK1/2 activity can be associated with upregulation of proapoptotic members of the Bcl-2 family, such as Bax [47–49]. Moreover, ERK activity has been shown to directly affect mitochondrial function [46] by decreasing mitochondrial respiration [50, 51] and mitochondrial membrane potential [51, 52], causing mitochondrial membrane disruption and Cytochrome c release [52–54].

Interestingly, GPER silencing was not able to prevent G-1 induced ERK phosphorylation, underlying the existence of alternative targets for G-1. These targets, similarly to GPER, are able to activate ERK1/2 signaling, however for a prolonged period, and clearly deserve further investigation.

Other papers evidenced inhibitory effects exerted by G-1 on the growth of different tumor cell types in a GPER independent manner [55–57], but a precise mechanism has not been defined. Although further studies are needed to clarify the molecular mechanisms behind G-1-dependent effects, this molecule could be a viable alternative to the current limited treatment options and therapeutic efficacy for adrenocortical cancer.

In conclusion, we demonstrated that treatment of H295R cells with G-1 reduced tumor growth *in vitro* and *in vivo* through a mechanism involving not only GPER activation. G-1 clearly causes cell-cycle arrest at the G_2_ phase and apoptosis through a mechanism that requires sustained ERK1/2 activation. Our previously published results highlighting the ability of OHT, a known GPER agonist and ESR1 antagonist, to reduce ACC cell growth, together with the present findings indicating the inhibitory effects exerted by G-1, open up new perspectives for the development of therapies with molecules modulating estrogen receptors action for the treatment of ACC.

## MATERIALS AND METHODS

### Cell culture and tissues

H295R cells were obtained from Dr W.E. Rainey (University of Michigan at Ann Arbor, USA) [58]. Cells were cultured as previously described [9]. Cell monolayers were subcultured onto 100 mm dishes for phosphatase activity and laddering assay (8 × 10^6^ cells/plate), 60 mm dishes for protein and RNA extraction (4 × 10^6^ cells/plate) and 24 well culture dishes for proliferation experiments (2 × 10^5^ cells/well) and grown for 2 days. Prior to experiments, cells were starved overnight in DMEM/F-12 medium without phenol red and containing antibiotics. Cells were treated with (±)-1-[(3aR*, 4S*, 9bS*)-4-(6-Bromo-1, 3-benzodioxol-5-yl)-3a, 4, 5, 9b-tetrahydro-3*H*-cyclopenta[*c*]quinolin-8-yl]-ethanone (G-1) (1 μM) (Tocris Bioscience, Bristol, UK) in DMEM/F-12 containing FBS-DCC 2, 5% (fetal bovine serum dextran-coated charcoal-treated). Inhibitors PD98059 (PD) (10 μM) (Calbiochem, Merck KGaA, Darmstadt, Germany) was used 1 h prior to G-1. Adrenocortical tumors, removed at surgery, and normal adrenal cortex, macroscopically dissected from adrenal glands of kidney donors, were collected at the hospital-based Divisions of the University of Padua (Italy). Tissue samples were obtained with the approval of local ethics committees and consent from patients, in accordance with the Declaration of Helsinki guidelines as revised in 1983. Diagnosis of malignancy was performed according to the histopathologic criteria proposed by Weiss et al. [59] and the modification proposed by Aubert et al. [60]. Clinical data of the six ACC patients included in this study are shown in Table 1. Patient C6 terminated mitotane treatment six months after beginning of therapy for severe gastrointestinal side effects. Patients C1 and C2 were treated with chemotherapy EAP protocol (etoposide, doxorubicin, and cisplatin) + mitotane.

**Table 1:** Clinical data of the 6 ACC patients analyzed in this study

Sample ID	Age(years)	Gender	Stage at surgery	Syndrome	Weiss score	Size (cm)	Outcome
C1	41	M	IV	Cushing	9	16	Died, 1 year
C2	17	F	IV	Cushing	9	14	Died, 18 months
C3	43	F	III	None	4	9	Died, 8 years
C4	46	M	III	None	3	18	Remission, 7 years
C5	47	M	IV	Cushing	9	14	Died, 1 year
C6	57	M	II	SubclinicalCushing	5	14	Remission, 4 years

### RNA extraction, reverse transcription and real time PCR

TRizol RNA isolation system (Invitrogen, Carlsbad, CA, USA) was used to extract total RNA from H295R, SKBR3 and ACCs. Each RNA sample was treated with DNase I (Invitrogen), and purity and integrity of the RNA were confirmed spectroscopically and by gel electrophoresis before use. One microgram of total RNA was reverse transcribed in a final volume of 30 μl using the ImProm-II Reverse transcription system kit (Promega Italia S.r.l., Milano, Italia); cDNA was diluted 1:2 in nuclease-free water, aliquoted, and stored at − 20°C. The nucleotide sequences for GPER amplification were forward, 5′-CGCTCTTCCTGCAGGTCAA-3′, and reverse, 5′-ATGTAGCGGTCGAAGCTCATC-3′ ; the nucleotide sequences for GAPDH amplification were forward, 5′-CCCACTCCTCCACCTTTGAC-3′, and reverse, 5′-TGTTGCTGTAGCCAAATTCGTT-3′. PCR reactions were performed in the iCycler iQ Detection System (Bio-Rad Laboratories S.r.l., Milano, Italia) using 0.1 μmol/L of each primer, in a total volume of 30 μl reaction mixture following the manufacturer's recommendations. SYBR Green Universal PCR Master Mix (Bio-Rad) with the dissociation protocol was used for gene amplification; negative controls contained water instead of first-strand cDNA. Each sample was normalized to its GAPDH content. The relative gene expression levels were normalized to a calibrator (normal tissue for ACC tissues or SKBR3 for H295R cells). Final results were expressed as n-fold differences in gene expression relative to GAPDH and calibrator, calculated using the ΔΔCt method as previously shown [61].

### Western blot analysis

Fifty μg of protein was subjected to western blot analysis [62]. Blots were incubated overnight at 4°C with antibodies against GPER, Cyclin E (CCNE), Cyclin B1 (CCNB1), phospho-Rb, Cytochrome c, Bax, Bcl-2, Parp1, pERK1/2-ERK2 (all from Santa Cruz Biotechnology, Santa Cruz CA, USA). Membranes were incubated with horseradish peroxidase (HRP)-conjugated secondary antibodies (Amersham Pharmacia Biotech, Piscataway, NJ) and immunoreactive bands were visualized with the ECL western blotting detection system (Amersham Pharmacia Biotech, Piscataway, NJ). To assure equal loading of proteins, membranes were stripped and incubated overnight with Glyceraldehyde 3-phosphate dehydrogenase (GAPDH) antibody (Santa Cruz Biotechnology).

### Histopathological analysis

Tumors were fixed in 4% formalin, sectioned at 5 μm and stained with hematoxylin and eosin, as suggested by the manufacturer (Bio-Optica, Milan, Italy).

### Immunohistochemical analysis

Paraffin-embedded sections, 5 mm thick, were mounted on slides precoated with poly-lysine, and then they were deparaffinized and dehydrated (seven to eight serial sections). Immunohistochemical experiments were performed as described [63], using mouse monoclonal Ki-67 primary antibody at 4°C over-night (Dako Italia Spa, Milano, Italy). Then, a biotinylated goat-anti-mouse IgG was applied for 1 h at room temperature, to form the avidin biotin-horseradish peroxidase complex (Vector Laboratories, CA, USA). Immunoreactivity was visualized by using the diaminobenzi-dine chromogen (Vector Laboratories). Counterstaining was carried out with hematoxylin (Bio-Optica, Milano, Italy). The primary antibody was replaced by normal rabbit serum in negative control sections.

### Cytochrome c detection

Cells were treated for 24 h, fractioned and processed for Cytochrome c detection as previously reported [26]. Briefly, cells were harvested by centrifugation at 2500 rpm for 10 min at 4°C. Pellets were resuspended in 50 μl of sucrose buffer (250 mM sucrose; 10 mM Hepes; 10 mM KCl; 1.5 mM MgCl2; 1 mM EDTA; 1 mM EGTA) (all from Sigma-Aldrich, Milano, Italy) containing 20 μg/ml aprotinin, 20 μg/ml leupeptin, 1 mM PMSF and 0.05% digitonine (Sigma-Aldrich). Cells were incubated for 20 min at 4°C and then centrifuged at 13,000 rpm for 15 min at 4°C. Supernatants containing cytosolic protein fraction were transferred to new tubes and the resulting mitochondrial pellets were resuspended in 50 μl of lysis buffer (1% Triton X-100; 1 mM EDTA; 1 mM EGTA; 10 mM Tris-HCl, pH 7.4) (all from Sigma-Aldrich) containing 20 μg/ml aprotinin, 20 μg/ml leupeptin, 1 mM PMSF (Sigma-Aldrich) and then centrifuged at 13,000 rpm for 10 min at 4°C. Equal amounts of proteins were resolved by 11% SDS/polyacrylamide gel as indicated in the Western blot analysis paragraph.

### Cell cycle analysis and evaluation of cell death

Subconfluent monolayers growing in 60 mm plates were depleted of serum for 24 h and treated for an additional 24 h with G-1. The cells were harvested by trypsinization and resuspended with 0.5 ml of Propidium Iodide solution (PI) (100 μg/ml) (Sigma-Aldrich) after treatment with RNase A (20 μg/ml). The DNA content was measured using a FACScan flow cytometer (Becton Dickinson, Mountain View, CA, USA) and the data acquired using CellQuest software. Cell cycle profiles were determined using ModFit LT program. Subconfluent monolayers growing in 60 mm plates were depleted of serum for 24 h and treated for 24 and 48 h with G-1. Trypsinized cells were incubated with Ligation Buffer (10 mM Hepes (pH = 7.4), 150 mM NaCl, 5 mM KCl, 1 mM MgCl2 and 1.8 mM CaCl2) containing Annexin-V-FITC (1:5000) (Santa Cruz) and with Propidium Iodide. Twenty minutes post-incubation at room temperature (RT) protected from light, samples were examined in a FACSCalibur cytometer (Becton Dickinson, Milano, Italy). Results were analyzed using CellQuest program.

### Caspases 9 and 3/7 activity assay

H295R cells after treatments were subjected to caspases 9 and 3/7 activity measurement with Caspase-Glo 9 and 3/7 assay kits (Promega) and modified protocol. Briefly, the proluminescent substrate containing LEHD or DEVD sequences (sequences are in a single-letter amino acid code) are respectively cleaved by Caspases 9 and 3/7. After caspases cleavage, a substrate for luciferase (aminoluciferin) is released resulting in luciferase reaction luminescent signal production. Cells were trypsinized, harvested and then suspended in DMEM-F12 before being incubated with an equal volume of Caspase-Glo reagent (40 μl) at 37°C for 1 h. The luminescence of each sample was measured in a plate-reading luminometer (Gen5 2.01) with Synergy H1 Hybrid Reader.

### TUNEL (terminal deoxynucleotidyltransferase-mediated dUTP nick-end labelling) assay

Cells were grown on glass coverslips, treated for 24 h and then washed with PBS and fixed in 4% formaldehyde for 15 min at room temperature. Fixed cells were washed with PBS and then soaked for 20 min with 0.25% of Triton X-100 in PBS. After two washes in deionized water, they were stained using the Click-iT^®^ TUNEL Alexa Fluor^®^ Imaging Assay (Invitrogen) according to the manufacturer's protocol. Co-staining with Hoechst33342 was performed to analyze the nuclear morphology of the cells after the treatment. Cell nuclei were observed and imaged under an inverted fluorescence microscope (200X magnification).

### Determination of DNA fragmentation

To determine the occurrence of DNA fragmentation, total DNA was extracted from control and G-1 (1 μM) treated (48 h) cells as previously described [26]. Equal amounts of DNA were analyzed by electrophoresis on a 2% agarose gel stained with Ethidium Bromide (Sigma-Aldrich).

### Assessment of cell proliferation

#### [^3^H]Thymidine incorporation assay

H295R cell proliferation after G-1 treatment was directly evaluated after a 6 h incubation with 1 μCi of [^3^H]thymidine (Perkin-Elmer Life Sciences, Boston, MA, USA) per well as previously described [64]. Each experiment was performed in triplicate and results are expressed as percent (%) of basal.

#### MTT assay

The effect of G-1 on cell viability was measured using 3-[4, 5-Dimethylthiaoly]-2, 5-diphenyltetrazolium bromide (MTT) assay as previously described [7]. Briefly, cells were treated for different times as indicated in figure legends. At the end of each time point fresh MTT (Sigma-Aldrich), re-suspended in PBS, was added to each well (final concentration 0.33 mg/ml). After 30 minutes incubation, cells were lysed with 1 ml of DMSO (Sigma-Aldrich). Each experiment was performed in triplicate and the optical density was measured at 570 nm in a spectrophotometer.

### Gene silencing experiments

For the gene silencing experiments, cells were plated in 12 well plates (1 × 10^5^ cells/well) for proliferation experiments or in 6 well plates (2 × 10^5^ cells/well) for Western blot analysis; cells were transfected with control vector (shRNA) or shGPER in 2, 5% DCC-FBS medium using lipofectamine 2000 transfection reagent (Invitrogen) according to the manufacturer's recommendations for a total of 72 h. For proliferation experiments cells were transfected for 24 h and then treated for 48 h before performing MTT assay.

### Xenograft model

Four-week-old nu/nu − Forkhead box N1^nu^ female mice were obtained from Charles River Laboratories Italia (Calco, Lecco, Italy). All animals were maintained in groups of five or less and quarantined for two weeks. Mice were kept on a 12 h/12 h light/dark regimen and allowed access to food and water *ad libitum*. H295R cells, 6 × 10^6^, suspended in 100 μl PBS (Dulbecco's Phosphate Buffered Saline), were combined with 30 μl of Matrigel (4 mg/ml) (Becton Dickinson) and injected subcutaneously in the shoulder of each animal. Resulting tumors were measured at regular intervals using a caliper, and tumor volume was calculated as previously described [65], using the formula: *V* = 0.52 (*L* × *W*^ 2^), where *L* is the longest axis of the tumor and *W* is perpendicular to the long axis. Mice were treated 21 days after cell injection, when tumors had reached an average volume of about 200 mm^3^. Animals were randomly assigned to be treated with vehicle or G-1 (Tocris Bioscience) at a concentration of 2 mg/kg/daily. Drug tolerability was assessed in tumor-bearing mice in terms of: a) lethal toxicity, i.e. any death in treated mice occurring before any death in control mice; b) body weight loss percentage = 100 − [(body weight on day x/body weight on day 1) × 100], where x represents a day during the treatment period [66, 67]. Animals were sacrificed by cervical dislocation 42 days after cell injection. All animal procedures were approved by Local Ethics Committee for Animal Research.

### *In vivo* magnetic resonance analyses

Mice were anesthetized with 1–2% isofluorane in O2, 1 L/min (Forane, Abbott SpA, Latina, Italia) and underwent MRI/MRS study. MR analyses were performed at 4.7 T on Agilent Technologies system (Palo Alto, CA, USA). T2-weighted MRI was acquired using a spin echo sequence with the following parameters: TR/TE = 3000/70 ms, section thickness of 1.0 mm, number of acquisitions = 4, point resolution of 256 μm.

### Scoring system

The immunostained slides of tumor samples were evaluated by light microscopy using the Allred Score [68] which combines a proportion score and an intensity score. A proportion score was assigned representing the estimated proportion of positively stained tumor cells (0 = none; 1 = 1/100; 2 = 1/100 to < 1/10; 3 = 1/10 to < 1/3; 4 = 1/3 to 2/3; 5 = > 2/3). An intensity score was assigned by the average estimated intensity of staining in positive cells (0 = none; 1 = weak; 2 = moderate; 3 = strong). Proportion score and intensity score were added to obtain a total score that ranged from 0 to 8. A minimum of 100 cells were evaluated in each slide. Six to seven serial sections were scored in a blinded manner for each sample.

### Data analysis and statistical methods

All experiments were performed at least three times. Data were expressed as mean values + standard error (SE), statistical significance between control (basal) and treated samples was analyzed using GraphPad Prism 5.0 (GraphPad Software, Inc.; La Jolla, CA) software. Control and treated groups were compared using the analysis of variance (ANOVA) with Bonferroni or Dunn's post hoc testing. A comparison of individual treatments was also performed, using Student's *t* test. Significance was defined as *p* < 0.05.
